# The follow-up of the robotic-assisted Soave procedure for Hirschsprung’s disease in children

**DOI:** 10.1007/s11701-021-01238-z

**Published:** 2021-04-11

**Authors:** Tran Anh Quynh, Pham Duy Hien, Le Quang Du, Le Hoang Long, Nguyen Thi Ngoc Tran, Tran Hung

**Affiliations:** Vietnam National Children’s Hospital, No 18, Alley 879, La Thanh, Dong Da, Hanoi Vietnam

**Keywords:** Hirschsprung’s disease, Robotic-assisted colon pull-through, Short rectal seromuscular sleeve

## Abstract

Robotic surgery offers three-dimensional visualization and precision of movement that could be of great value to gastrointestinal surgeons. There were many previous reports on robotic technology in performing Soave colonic resection and pull-through for Hirschsprung’s disease in children. This study described the follow-up of the Robotic-assisted Soave procedure for Hirschsprung’s disease in children. Robotic-assisted endorectal pull-through was performed using three robotic arms and an additional 5-mm trocar. The ganglionic and aganglionic segments were initially identified by seromuscular biopsies. The rest of the procedure was carried out according to the Soave procedure. We left a short rectal seromuscular sleeve of 1.5–2 cm above the dentate line. From December 2014 to December 2017, 55 pediatric patients were operated on. Age ranged from 6 months to 10 years old (median = 24.5 months). The aganglionic segment was located in the rectum (*n* = 38), the sigmoid colon (*n* = 13), and the left colon (*n* = 4). The mean total operative time was 93.2 ± 35 min (ranging from 80 to 180 min). Minimal blood was lost during the surgery. During the follow-up period, 41 patients (74.6%) had 1–2 defecations per day, 12 patients (21.8%) had 3–4 defecations per day, and 2 patients (3.6%) had more than 4 defecations per day. Fecal incontinence, enterocolitis, and mild soiling occurred in three (5.4%), four (7.3%), and two pediatric patients, respectively. Robotic-assisted Soave procedure for Hirschsprung’s disease in children is a safe and effective technique. However, a skilled robotic surgical team and procedural modifications are needed.

## Background

Hirschsprung’s disease (HD) is a common cause of intestinal obstruction in the newborn. It is characterized by the absence of ganglionic cells in the distal bowel beginning at the internal sphincter and extending proximally to varying distances [1]. Since the first successful treatment was reported in 1948 [2], several pull-through techniques have been developed including the laparoscopic colon pull-through which was performed by Soave and Duhamel [3, 4]. This procedure was introduced in 1994 for HD [5]. Since then, various laparoscopic techniques have been used [5–7], of which, Georgeson’s technique has become the most popular [7].

Currently, robotic surgery outweighs laparoscopic surgery due to its advantages of superior visualization and dexterity, tremor reduction, as well as a faster learning curve [8–13]. In 2011, Hebra et al. had the first report of Robotic Swenson pull-through for HD in twelve infants [11]. Rickey et al. (2013) reported a robotic-assisted Soave procedure in a young adult [12], and Mattiolo et al. (2017) had applied this technique for older children or adults with HD [13].

All minimally invasive procedures (either laparoscopic or not) include an extended transanal endorectal dissection that can traumatize the sphincteric structures, rendering the prolonged and tough anal dilatations. However, patients with higher age required more extensive efforts during transanal endorectal dissections for peritoneal reflection because they were more likely to experience the increase of sphincter structure damages. Robotic surgery might be the potential to overcome this barrier. In this study, we decided to apply the robot-assisted endorectal dissection resorting to the very first principles of dissection reported by Franco Soave, and report early outcomes of the Robotic-assisted Soave procedure for Hirschsprung’s disease in children.

## Material and method

### Study design and sampling method

From December 2014 to December 2017, 55 patients were operated on by the same surgeons. Patients whose ages ranged from 6 months to 10 years old (Median 24.5 months) with biopsy documented distal colon aganglionosis were treated with Robotic-assisted colonic resection and pull-through using The Da Vinci^®^ robotic system (Intuitive Surgical, Inc., Sunnyvale, CA).

Preoperative diagnosis was based on typical clinical manifestations (constipation, requiring regular enemas) with X-ray findings of the narrow distal segment, a transitional zone, and upper proximal dilated segment detected on contrast studies of the rectum and colon. Ten patients had a preoperative colostomy and forty-five patients were without colostomy. The diagnosis was confirmed by intraoperative frozen biopsy and reconfirmed by postoperative conventional histopathological study. Preparation of the colon was performed by colonic irrigation with normal saline for 7 days. An oral antibiotic regimen of metronidazole was given for 3 days preoperatively. Also, third-generation cephalosporin and aminoglycoside were given during anesthesia induction and continued for 6 days.

### Robotic surgical technique

A 12-mm trocar was put via a two-cm incision in the umbilical area. Two 8-mm trocars were inserted in the right iliac fossa and the left flank. Robotic arms were inserted in these three trocars and a further 5-mm trocar was inserted in the right hypochondria to assist traction and manipulation with conventional laparoscopic instruments. CO_2_ insufflation was maintained at a pressure of 12 mmHg.

A seromusculature biopsy was taken for a frozen section from suspected aganglionic and ganglionic segments. A window on the sigmoid mesentery was created, then dissection around the rectal wall was performed circumferentially down to the pelvis under the peritoneal reflection approximately 2 cm anteriorly and the level of the coccyx posteriorly. The sigmoid artery trunk was clipped then divided. The mesentery was mobilized up to the level of the inferior mesenteric artery.

A surgical lone star retractor (manufactured by Lone Star, medical products, 11211 Cash Road, Stanford, Tex) was used to expose the anus for the transanal dissection. A circumferential incision was made in the mucosa at 0.5–1 cm proximal to the dentate line. The upward submucosal dissection was carried out for approximately 6 cm. The seromuscular layer of the anterior rectal wall was pulled down and divided longitudinally. This layer was then incised circumferentially to free the rectum completely. The seromuscular sleeve was removed leaving a cuff of 1.5–2 cm in length from the dentate line.

Next, the colon was pulled through the anus. The aganglionic and dilated segments were resected. The coloanal anastomosis was fashioned manually 0.5–1 cm above the dentate line. Oral intake of clear fluid was initiated 12 h after the operation and advanced to the formula on the second day. Anal dilatation was begun at home 15 days after the operation and continued for 3 months in all patients. Follow-up was scheduled at 3 weeks after the date of operation and then at regular 3–6-month intervals. The colostomy was closed 2 months after the robotic-assisted colon pull-through.

## Results

There were 44 boys and 11 girls with ages ranging from 6 months to 10 years old (median 24.5 months). The aganglionic segment was located in the rectum (*n* = 38), the sigmoid colon (*n* = 13), and the left colon (*n* = 4). The length of the resected bowel ranged from 10 to 35 cm (Table 1).

**Table 1:** Ages and length of resected bowel

	Length of resected bowel			Total
	< 15 cm	16–30 cm	> 30 cm	
Ages				
< 24 months	22	13	0	35
25–60 months	9	7	1	17
> 60 months	0	1	2	3
Total	31	21	3	55

### Operative data and postoperative outcomes

The operation times are summarized in Table 2. There was no conversion to laparoscopic surgery or laparotomy and no intraoperative complications, including injury of other organs. There was minimal blood loss during the surgery. The primary coloanal anastomosis was carried out in all patients. Postoperative hospitalization ranged from 4 to 8 days (mean = 5.5 days).

**Table 2:** Operative time (minutes)

	Minimum	Maximum	Mean
Docking time	5	25	11.5 ± 4
Console time	35	110	57.7 ± 21
Anastomosis time	25	40	31.3 ± 15
Total operative time	80	180	93.2 ± 35

Follow-up was obtained in 55 patients (100%) with a follow-up ranging from 30 to 66 months (median = 43.2 months). All these patients responded well to anal dilatation, no anastomotic stenosis. No patient had urinary incontinence. Erectile function, evaluated by the patient’s parents, was present in all boys. 96.4% of patients had 1–4 bowel movements per day. Enterocolitis and mild soiling occurred in four (7.3%), and two pediatric patients, respectively (Table 3).

**Table 3:** Bowel movement

Bowel movement	Number	Percentage
Number of defecations/day		
1–2	41	74.6
3–4	12	21.8
> 4	2	3.6
Incontinence	0	0
Constipation	0	0
Enterocolitis	4	7.3
Mild soiling	2	3.6

## Discussion

The primary laparoscopic-assisted endorectal pull-through using a variation of the Soave procedure was adopted by many surgeons as the procedure of choice for the treatment of HD [7]. It provides a clear delineation of pelvic structures and better cosmetic results. The intestinal function is minimally disturbed. Oral feeding can be resumed soon after an operation. Laparoscopy can determine the transition zone histologically and can also avoid twisting, bleeding, and tearing in the mesenteric vessels.

Robotic surgery is introduced to overcome some limitations represented by limited operative space and poor visualization of the deep pelvis and the limited degree of freedom of conventional laparoscopy. With specific regard to HD, the major strength of robotic surgery is the possibility to perform an extended seromuscular dissection thanks to a better 3D visualization of the robotic camera and to the degrees of freedom of robotic instruments that allow an ideal identification and dissection of the submucosa. These advantages are well evident if we compare robotic surgery either with laparoscopic surgery or with conventional open surgery which has limited working space, such as that of the deep pelvis. An extensive intra-abdominal seromuscular dissection affects the need for a far less extensive transanal dissection and may potentially reduce the risk for complications related to the perineal steps. Reduced sphincters stretching and less endorectal dissection are required in robotic surgery compared to conventional laparoscopic surgery. Moreover, easier seromuscular dissection can be performed down to 2–3 cm above the pectinate line in robotic surgery [11, 13]. The disadvantages of robotic surgery are well documented. The robot itself costs more than one million dollars, not including the maintenance contract and expensive disposable equipment [8–11].

To the best of our knowledge, our study is the largest series of robotic-assisted surgery for Hirschsprung’s disease in infants. The total operative time was shorter than previous reports, especially in the robotic console component (mean, 93.2 min; range: 80 – 180 min; mean console time, 57.7 min) [11, 13]. The factor that can explain this difference is that our department has a lot of patients with HD and we are trained and operated to use proficient techniques in open surgery, laparoscopic surgery, so when conducted robot surgery, our operative time was shorter than other authors with the advantages of robot surgery. In the study of Liem N.T et al. (2009) the mean operative time was 152 min (200 patients with Hirschsprung’s disease) [14], and report of Hau B.D et al. (2011), the median operative time (134 min, 47 patients) longer than our research [15].

Our results showed that robotic-assisted colon pull-through was associated with a low rate of postoperative complications. Early anastomotic leakage was not encountered in our series. It has been seen in 5.6–11.2% of cases in some series when using open surgery [16, 17]. This complication also occurred in 1.5–2.9% of cases when using the transanal approach [18, 19], and the laparoscopic surgery had anastomotic fistula occurred 1% [14]. Primary anastomosis has been performed safely in all patients. However, the colon stump should be left and removed in a second operation if anastomotic security is not ensured.

No anastomotic stenosis was seen in our series. This rate compares to what occurred in that reported for open surgery or the transanal approach [16, 20]. We had no cases of rectal stenosis requiring daily dilatation.

A short cuff could be an important factor in reducing severe rectal stenosis and enterocolitis. In the transanal pull-through, Nasr and Langer also noticed that the incidence of enterocolitis and rectal stenosis requiring daily dilatation decreased in the short cuff group, in comparison with the long-cuff group [21]. In the robotic-assisted colon pull-through, one can remove not only the aganglionic segment and transitional segment, but also the malfunctioned dilated segment because the length of colonic mesentery vessels is always sufficient. This may further reduce the rate of postoperative enterocolitis. The rate of enterocolitis in our series was 7.3%, compared with 9.5% of Liem’s report [14], and 30% of Nasr’s report [21].

The defecation function was satisfactory in long-term follow-up. 74.6% of patients had 1–2 bowel movements per day. All patients maintained urinary continence. The erectile function was not impaired in male patients reflecting good protection of the pelvic nervous system in our robotic-assisted colon pull-through. The dissection close to the rectal wall was mandatory to avoid injury to adjacent structures.

Besides these advantages, the surgical robot also has disadvantages, including the expensive purchase price of the robot, cost of surgical instruments, and complete lack of haptic feedback.

Currently, robotic surgery is only used for selected patients and some pediatric surgery centers. We hope that shortly, robotic surgery will be widely used with technical refinements, further miniaturization of robotic instruments, and reducing instrument costs.

## Conclusion

In conclusion, the robotic-assisted Soave procedure for Hirschsprung’s disease in children is safe and effective. Future studies are needed to determine the long-term outcomes of this approach and possibly determine its superiority over conventional laparoscopic procedures.

## References

[1] Tomuschat C, Puri P (2015). *RET* gene is a major risk factor for Hirschsprung’s disease: a meta-analysis. PediatrSurgInt.

[2] Swenson O, Bill AH (1948). Resection of rectum and rectosigmoid with preservation of the sphincter for benign spastic lesions producing megacolon; an experimental study. Surgery.

[3] Soave F (1964). Hirschsprung’s disease: a new surgical technique. Arch Dis Child.

[4] Duhamel B (1960). A new operation for the treatment of Hirschsprung’s disease. Arch Dis Child.

[5] Smith BM, Steiner RB, Lobe TE (1994). Laparoscopic Duhamel pull-through procedure for Hirschsprung’s disease in childhood. J LaparoendoscSurg.

[6] George C, Hammes M, Schwarz D (1995). Laparoscopic Swenson pull-through procedure for congenital megacolon. AORN J.

[7] Georgeson KE, Fuenfer MM, Hardin WD (1995). Primary laparoscopic pull-through for Hirschsprung’s disease in infants and children. J PediatrSurg.

[8] Van Haarsteren G, Levine S, Hayes W (2009). Pediatric robotic surgery: early assessment. Pediatrics.

[9] Najmaldin A, Dasgupta P, Fitzpatrick J, Kirby R (2010). Principles in robotic-assisted surgery in children. New technologies in urology.

[10] Peter CA (2009). Pediatric robotic surgery: too early an assessment?. Pediatric.

[11] Hebra A, Smith VA, Lesher AP (2011). Robotic Swenson pull-through for Hirschsprung’s disease in infants. Am Surg.

[12] Rickey J, Robinson CC, Camps JI, Lagares-Garcia JA (2013). Robotic-assisted Soave procedure in an 18-year-old man with adult short-segment Hirschsprung’s disease. Am Surg.

[13] Mattioli G, Pio L, Leonelli L, Razore B, Disma N, Montobbio G, Jasonni V, Petralia P, Pini Prato A (2017). A provisional experience with robot-assisted soave procedure for older children with hirschsprung disease: back to the future?. J LaparoendoscAdvSurg Tech.

[14] Liem NT, Hau BD, Quynh TA (2009). Early and late outcomes of primary laparoscopic endorectal colon pull-through leaving a short rectal seromuscular sleeve for Hirschsprung disease. J PediatrSurg.

[15] Hau BD, Quynh TA, Anh VH (2011). Early and late outcomes of primary laparoscopic endorectal colon pull-through leaving a short rectal seromuscular sleeve for Hirschsprung disease. J LaparoendoscAdvSurg Tech.

[16] Sherman JO, Snyder ME, Weitzman JJ (1989). A 40-year multinational retrospective study of 880 Swenson procedures. J PediatrSurg.

[17] Kleinhaus S, Boley SJ, Sheran M (1979). Hirschsprung’s disease. A survey of the surgical section of the America academy of pediatrics. J PediatrSurg.

[18] Hadidi A (2003). Transanalendorectal pull-through for Hirschsprung’s disease: experience with 68 patients. J PediatrSurg.

[19] Podevine G, Lardy H, Azzis U (2006). Technical problem and complications of a transanal pull-through for Hirschprung’s disease. Eur J PediatrSurg.

[20] Elhalaby EA, Hashish A, Elbarbary MM (2004). Transanal one-stage endorectal pull-through for Hirschsprung’s disease: a multicenter study. J PediatrSurg.

[21] Nasr A, Langer JC (2007). Evolution of the technique in the transanal pull-through for Hirschsprung’s disease: effect on the outcome. J PediatrSurg.

